# Silicea

**Published:** 1896-06

**Authors:** 


(Translated by the late Dr. Samuel Lilienthal.)

### SILICEA.

(Translated by the late Dr. Samuel Lilienthal.)

An old man of seventy complains of right-sided rheumatic pains in his face and palate; worse in damp weather, when the cheek swells up and reddens. Even touching the painful spots with his tongue causes excrutiating pains. Considering it as affection of the periosteum, Goullon prescribed Silicea$^{30}$, four drops in half a glass of water, thrice daily a teaspoonful, which not only quickly removed all pain, but also cleared up his murky urine, of which he had complained for some time.—*Pop. Zeitschrift*, 16, 91.

